# Association between Lipoprotein(a) and diet quality with cardiovascular disease risk: the multi-ethnic study of atherosclerosis

**DOI:** 10.1016/j.ajpc.2026.101627

**Published:** 2026-04-09

**Authors:** Amier Haidar, Harveen K. Sekhon, Rishi Rikhi, Karol E. Watson, Michael D. Shapiro

**Affiliations:** a Division of Cardiovascular Medicine, McGovern Medical School, University of Texas Health Science Center at Houston, USA; b Division of Cardiology, David Geffen School of Medicine University of California, Los Angeles, USA; c Center for Prevention of Cardiovascular Disease, Section on Cardiovascular Medicine, Department of Internal Medicine, Wake Forest University School of Medicine, Winston-Salem, North Carolina, USA

**Keywords:** Lipoprotein(a), Diet quality, Cardiovascular disease risk, Prevention

## 1 Introduction

Lipoprotein(a) [Lp(a)] is a genetically determined, causal risk factor for atherosclerotic cardiovascular disease (ASCVD), with risk increasing across its distribution [1,2]. Current guidelines recommend measurement of Lp(a) to identify individuals at elevated lifetime risk, while several Lp(a)-targeted treatments are currently being investigated, there are no approved Lp(a) lowering therapies currently [1,3]. Intensive risk factor control is warranted in individuals with high Lp(a) with the aim of reducing overall cardiovascular risk [1]. While dietary modifications are ineffective at lowering Lp(a) levels, the impact of a healthy or unhealthy diet on an individual’s absolute ASCVD risk among those with high Lp(a) concentrations remain unknown [1,2]. The objective of this study is to examine the joint association between diet quality, Lp(a), and incident ASCVD risk in a large multi-ethnic primary prevention cohort.

## 2 Methods

The Multi-Ethnic Study of Atherosclerosis (MESA) is a prospective cohort of 6814 adults aged 45–84 years without baseline cardiovascular disease, enrolled from six U.S. sites between 2000 and 2002; study design and protocols have been previously described [4]. Demographic, clinical, and laboratory data were collected using standardized methods, including assessment of lipids, glucose, blood pressure, medications, smoking, physical activity, and anthropometric measures. All participants provided written informed consent, and the study protocol was approved by the institutional review boards at each participating institution.

Diet was assessed at baseline using a validated 120-item food-frequency questionnaire, as previously described [4]. Diet quality was evaluated using the Alternative Healthy Eating Index (AHEI), with participants in the top quintile classified as having a healthy diet. Hard CVD events included non-fatal myocardial infarction, resuscitated cardiac arrest, fatal coronary heart disease (CHD), stroke (not TIA), and death resulting from stroke. Detailed adjudication methods have been previously reported [4].

The study population was divided into four groups: Group 1: Lp(a) <50mg/dL with a healthy diet, Group 2: Lp(a) <50mg/dL without a healthy diet, Group 3: Lp(a) ≥50mg/dL with a healthy diet, and Group 4: Lp(a) ≥50mg/dL without a healthy diet. Cox proportional hazards (CPH) models were used to evaluate the association between Lp(a), diet, and ASCVD events. Model 1 was unadjusted, Model 2 was adjusted for age, gender, and race. Model 3 included model 2 plus tobacco use and physical activity. A model that included interactions for diet quality and Lp(a) was assessed. The proportional hazards assumption was assessed using Schoenfeld residuals, and no significant violations were observed. Competing risks regression was performed using Fine–Gray hazard models, treating non-cardiovascular death as a competing event. Sensitivity analyses were conducted to address potential reverse causation by excluding participants with ASCVD events occurring within the first year of follow-up. STATA 18.5 was used for all analyses.

## 3 Results

A total of 6400 participants were included (mean age 62.3 ± 10.2 years; 52% female). Of these, 1274 (20%) had elevated Lp(a) concentrations (≥50 mg/dL), and 1284 (20%) had healthy diets. Baseline characteristics across the four joint Lp(a)–diet groups are shown in Table 1. Traditional cardiovascular risk factors, including systolic blood pressure, diabetes, BMI, and physical activity were broadly similar across diet–Lp(a) combinations.

**Table 1:** Baseline characteristics of Lp(a) and diet quality groups.

Baseline Characteristics	Group 1: Lp(a) <50 mg/dL and healthy diet (n = 1024)	Group 2: Lp(a) <50 mg/dL and unhealthy diet (n = 4102)	Group 3: Lp(a) ≥50 mg/dL and healthy diet (n = 260)	Group 4: Lp(a) ≥50 mg/dL and unhealthy diet (n = 1014)
Age	64.6 (10.0)	61.5 (10.3)	63.9 (10.2)	62.1 (10.0)
Female	650 (63.5)	1960 (47.8)	192 (73.8)	566 (55.8)
White	416 (40.6)	1689 (41.2)	90 (34.6)	304 (30.0)
Chinese American	204 (19.9)	513 (12.5)	24 (9.2)	50 (4.9)
Black	209 (20.4)	890 (21.7)	110 (42.3)	489 (48.2)
Hispanic	195 (19.1)	1010 (24.6)	36 (13.8)	171 (16.9)
Lp(a), mg/dL	16.6 (12.7)	15.8 (12.3)	86.9 (35.9)	86.1 (33.9)
AHEI	76.7 (5.2)	57.9 (7.9)	77.0 (5.7)	57.8 (8.3)
Tobacco use (pack-years)	8.7 (17.8)	11.9 (22.0)	7.2 (14.8)	11.3 (19.6)
Total Moderate and Vigorous Physical Activity (Met-Min/Week)	5527 (5495)	5718 (6030)	5495 (4932)	5989 (5833)
HDL Cholesterol (mg/dL)	53.2 (15.5)	49.6 (14.3)	56.1 (15.1)	53.1 (15.4)
LDL Cholesterol (mg/dL)	113.1 (29.3)	115.2 (31.0)	125.6 (30.9)	126.3 (32.5)
Systolic Blood Pressure (mmHg)	127.5 (22.0)	125.6 (20.9)	125.8 (22.4)	128.7 (21.9)
Lipid Lowering therapy	158 (15.4)	617 (15.1)	56 (21.6)	212 (20.9)
Diabetes	115 (11.2)	499 (12.2)	33 (12.7)	145 (14.3)
BMI, kg/m2	27.1 (4.9)	28.4 (5.4)	27.9 (5.4)	28.9 (5.6)

Continuous variables reported as mean (SD). Categorical variables displayed as total count (%). BMI indicates body mass index; HDL-C, high-density lipoprotein-cholesterol; LDL-C, low-density lipoprotein-cholesterol; Lp(a), lipoprotein(a); AHEI, Alternative Healthy Eating Index.

Over a median follow-up of approximately 18 years, 766 hard ASCVD events occurred. Event rates and hazard ratios across groups are presented in Table 2. In unadjusted analyses, ASCVD incidence ranged from 7.7 to 10.0 per 1000 person-years, with the lowest event rate observed among participants with Lp(a) <50 mg/dL and a healthy diet (Group 1) and the highest among those with Lp(a) ≥50 mg/dL and an unhealthy diet (Group 4). Ten-year absolute ASCVD risk increased progressively across groups, from 4.2% in Group 1 to 5.9% in Group 4.

**Table 2:** Risk for cardiovascular disease events according to Lp(a) and diet quality groups.

Risk groups	Events/total (%)	Incidence rate (per thousand p-yrs)	10-year absolute risk (%)	HR (95% CI); *P* value (model 1)	HR (95% CI); *P* value (model 2)	HR (95% CI); *P* value (model 3) (primary model)	HR (95% CI); P value (model 4)
Group 1: Lp(a) <50 mg/dL and healthy diet	111/1024 (10.8)	7.7	4.2	1.00 (reference); NA	1.00 (reference); NA	1.00 (reference); NA	1.00 (reference); NA
Group 2: Lp(a) <50 mg/dL and unhealthy diet	485/4102 (11.8)	8.5	5.2	1.10 (0.89, 1.35); 0.356	1.24 (1.01, 1.53); 0.041	1.25 (1.01, 1.55); 0.036	1.22 (0.99, 1.51); 0.068
Group 3: Lp(a) ≥50 mg/dL and healthy diet	33/260 (12.7)	8.9	5.4	1.15 (0.78, 1.70); 0.468	1.24 (0.84, 1.84); 0.278	1.27 (0.86, 1.88); 0.226	1.30 (0.87, 1.94); 0.197
Group 4: Lp(a) ≥50 mg/dL and unhealthy diet	137/1014 (13.5)	10.0	5.9	1.29 (1.01, 1.66); 0.043	1.48 (1.14, 1.91); 0.003	1.47 (1.13, 1.90); 0.004	1.39 (1.07, 1.81); 0.015

Multivariable Cox proportional hazards model 1 unadjusted; model 2 adjusted for age, sex, race, and ethnicity; model 3 adjusted for model 2+pack years and physical activity; and model 4 adjusted for model 3+diabetes history, high-density lipoprotein cholesterol, low-density lipoprotein cholesterol, lipid lowering medication use, systolic blood pressure, diabetes medication use, waist-to-height ratio; HR, hazard ratio; Lp(a), lipoprotein(a); and P-yrs, person-years.

**Fig. 1:**
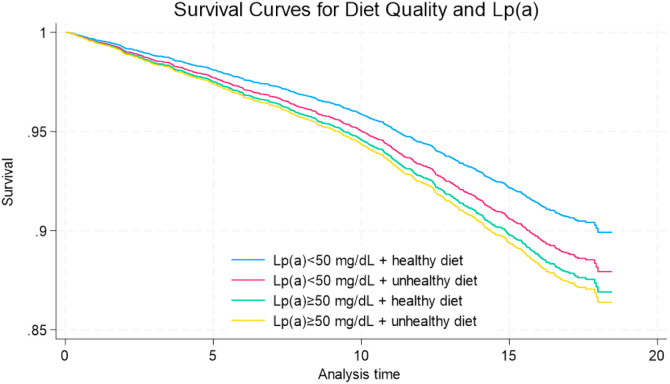
Survival curves for incident cardiovascular disease by joint categories of diet quality and lipoprotein(a). CVD-free survival was highest among individuals with Lp(a) <50 mg/dL and a healthy diet and lowest among those with Lp(a) ≥50 mg/dL and an unhealthy diet, demonstrating a graded decline in survival across groups.

In multivariable Cox models adjusted for age, sex, race/ethnicity, tobacco exposure, and physical activity (Model 3), both diet quality and Lp(a) were independently associated with ASCVD risk. Compared with participants in Group 1, those with low Lp(a) but an unhealthy diet had a 25% higher risk of ASCVD (HR 1.25; 95% CI 1.01–1.55). Participants with elevated Lp(a) and an unhealthy diet (Group 4) had the highest relative risk (HR 1.47; 95% CI 1.13–1.90). Among individuals with elevated Lp(a), adherence to a healthy diet did not fully mitigate the excess risk; Group 3 demonstrated a nonsignificant but directionally increased risk (HR 1.27; 95% CI 0.86–1.88). When modeled independently, Lp(a) ≥50 mg/dL was associated with a 20% higher risk of ASCVD events (HR 1.20; p = 0.044), while adherence to a healthy diet was associated with a 19% lower risk (HR 0.81; p = 0.027). No statistical interaction between diet quality and Lp(a) was observed, suggesting additive rather than multiplicative effects on ASCVD risk. Kaplan–Meier survival curves demonstrated clear separation among the four groups, with the most favorable event-free survival in individuals with low Lp(a) and a healthy diet, and the poorest survival in those with elevated Lp(a) and an unhealthy diet (Fig. 1).

## 4 Discussion

In this large, multi-ethnic cohort, both elevated Lp(a) and poor diet quality were independently associated with higher long-term ASCVD risk, with the greatest risk observed when both were present. Although a healthy diet was associated with lower absolute risk, it did not fully attenuate the excess risk associated with elevated Lp(a), supporting additive rather than synergistic effects.

These findings extend prior evidence by demonstrating that diet quality influences cardiovascular risk across all levels of Lp(a), despite the persistence of elevated risk among individuals with high Lp(a) [1,2]. This supports the concept of parallel, additive mechanisms in which Lp(a) confers a fixed baseline risk independent of behavioral pathways, while diet modifies risk through separate set of modifiable pathways including metabolic and inflammatory mechanisms. As a result, healthy dietary patterns may improve overall cardiometabolic risk but are unlikely to fully offset the hazard associated with elevated Lp(a).

Clinically, these results reinforce the importance of measuring Lp(a) to identify individuals at elevated lifetime risk and emphasize that lifestyle optimization remains critical even in the setting of genetic risk. Although lifestyle measures cannot reduce Lp(a), our results show that high-quality dietary patterns confer meaningful absolute risk reduction even among individuals with elevated Lp(a). The excess risk observed in individuals with elevated Lp(a) despite healthy dietary patterns highlights the need for comprehensive prevention strategies, including aggressive risk factor control and emerging Lp(a)-lowering therapies.

Strengths of this study include the large, diverse cohort, long follow-up, and rigorously adjudicated outcomes. Limitations include reliance on a single baseline dietary assessment and the relatively small number of participants with both elevated Lp(a) and a healthy diet, which may limit precision in subgroup analyses.

## 5 Conclusion

In summary, elevated Lp(a) and poor diet quality were independently associated with higher long-term ASCVD risk in a large multi-ethnic cohort. Healthy diet was linked to lower absolute risk but did not fully mitigate the increased hazard associated with genetically elevated Lp(a). These findings underscore the need for comprehensive cardiovascular prevention strategies in individuals with high Lp(a), including lifestyle optimization, aggressive risk factor control, and consideration of future Lp(a)-targeted pharmacologic therapies.

## Funding

This research was supported by contracts 75N92020D00001, HHSN268201500003I, N01-HC-95159, 75N92020D00005, N01-HC- 95160, 75N92020D00002, N01-HC-95161, 75N92020D00003, N01-HC-95162, 75N92020D00006, N01-HC-95163, 75N92020D00004, N01-HC-95164, 75N92020D00007, N01-HC-95165, N01-HC-95166, N01-HC-95167, N01-HC-95168, and N01-HC-95169 from the 10.13039/100000050National Heart, Lung, and Blood Institute and by grant Nos. UL1-TR-000040, UL1-TR-001079, and UL1-TR-001420 from the National Center for Advancing Translational Sciences. This publication was developed under the Science to Achieve Results research assistance agreements Nos. RD831697 (MESA Air) and RD-83,830,001 (MESA Air Next Stage) awarded by the US Environmental Protection Agency. It has not been formally reviewed by the US Environmental Protection Agency. The views expressed in this document are solely those of the authors, and the US Environmental Protection Agency does not endorse any products or commercial services mentioned in this publication.

## CRediT authorship contribution statement

**Amier Haidar:** Writing – review & editing, Writing – original draft, Visualization, Validation, Investigation, Formal analysis, Data curation, Conceptualization. **Harveen K. Sekhon:** Writing – review & editing, Conceptualization. **Rishi Rikhi:** Writing – review & editing, Validation. **Karol E. Watson:** Writing – review & editing, Supervision, Resources. **Michael D. Shapiro:** Writing – review & editing, Supervision, Conceptualization.

## Declaration of competing interest

The authors declare that they have no known competing financial interests or personal relationships that could have appeared to influence the work reported in this paper.
